# Intensity-Modulated Radiotherapy in Patients with Cervical Cancer. An intra-individual Comparison of Prone and Supine Positioning

**DOI:** 10.1186/1748-717X-5-63

**Published:** 2010-07-02

**Authors:** Carmen Stromberger, Yves Kom, Michael Kawgan-Kagan, Tristan Mensing, Ulrich Jahn, Achim Schneider, Volker Budach, Christhardt Köhler, Simone Marnitz

**Affiliations:** 1 Department of Radiooncology, Charité University Hospital, Campus CCM and CVK, Augustenburger Platz 1, 13353 Berlin, Germany; 2 Department of Gynaecology, Charité University Hospital, Campus CCM and CBF, Charitéplatz 1, 10117 Berlin, Germany

## Abstract

**Background:**

Chemoradiation for cervical cancer patients is associated with considerable gastrointestinal toxicity. Intensity-modulated radiotherapy (IMRT) has demonstrated superiority in terms of target coverage and normal tissue sparing in comparison to conventional 3D planning in gynaecological malignancies. Whether IMRT in prone (PP) or supine position (SP) might be beneficial for cervical cancer patients remains partially unanswered.

**Methods:**

10 patients on FIGO stage IB-III cervical cancer, 6 patients for definitive and 4 patients for adjuvant external beam pelvic RT, were planned in PP and SP using a 7-field IMRT technique. IMRT plans for PP and SP (mean dose, D_mean _50.4 Gy) were optimized in terms of PTV coverage (1^st ^priority) and small bowel sparing (2^nd ^priority). A comparison of DVH parameters for PTV, small bowel, bladder, and rectum was performed.

**Results:**

The comparison showed a similar PTV coverage of 95% of the prescribed dose and for target conformity in IMRT plans (PP, SP). PTV, rectum and bladder volumes were comparable for PP and SP. Significantly larger volumes of small bowel were found in PP (436 cc, + 35%, p = 0.01). PP decreased the volume of small bowel at 20-50.4 Gy (p < 0.05) and increased the rectum volumes covered by doses from 10-40 Gy (p < 0.01), the V50.4 was < 5% in both treatment positions. Bladder sparing was significant better at 50.4 Gy (p = 0.03) for PP.

**Conclusion:**

In this dosimetric study, we demonstrated that pelvic IMRT in prone position for patients with cervical cancer seems to be beneficial in reducing small bowel volume at doses ≥20 Gy while providing similar target coverage and target conformity. The use of frequent image guidance with KV (kilovolt) or MV (megavolt) computertomography can reduce set-up deviations, and treatment in prone position can be done with a higher set-up accuracy. Clinical outcome studies are needed to affirm lower toxicity.

## Background

Chemoradiation is the treatment of choice in locally advanced, lymph node positive and/or high-risk cervical cancer patients [1-9]. The treatment is associated with considerable gastrointestinal, genitourinary, and haematological toxicity [10,11]. Furthermore, the combination of radical hysterectomy and adjuvant radiation leads to an increase of therapy related toxicity [12,13]. In order to cover tumour and locoregional lymph nodes adequately with a 2-D or 3-D conformal radiotherapy technique, large portions of small bowel must be included in the radiation ports. The incidence and severity of gastrointestinal morbidity depends on the volume of small bowel and on the radiation dose to the small bowel and correlates with a pelvic surgery prior to radiotherapy [14-17]. In the past, efforts were made to reduce the incidence and severity of gastrointestinal toxicity. Pelvic radiotherapy in prone position on a belly-board device resulted in a significant sparing of small bowel [18-23]. A recent study showed that patient set-up in prone position is subject to larger systematic errors, but the set-up in supine position harbours larger random errors [24]. A superiority of intensity-modulated radiotherapy (IMRT) over conventional 3-D planning has been demonstrated for gynaecological malignancies in terms of target coverage and normal tissue sparing [25-29]. Furthermore, IMRT offers the possibility of dose escalation without increased therapy related toxicity [30,31]. Dose escalation with a simultaneous integrated boost is even feasible in patients with advanced cervical cancer [32]. Even though oncological results for IMRT seem to be similar to those for 3-D planning [33-35], issues concerning the optimal margins, tumour regression, movement of organs at risk during the course of radiotherapy and the optimal treatment position remain a challenging field of research. In this study, we assess whether a pelvic 7-field IMRT in prone or supine position can be more efficient regarding the sparing of small bowel, rectum and bladder in patients with cervical cancer.

## Methods

### Patients

10 patients with histological confirmed cervical cancer on FIGO stage IB1, IB2, IIB and IIIB were selected for this study. 6 patients were treated with definitive chemoradiation (FIGO stage: IB1 in 2 patients, IB2, IIA, IIB and IIIB each in one patient). All of these patients underwent transperitoneal laparoscopic pelvic and paraaortic lymphadenectomy as described previously [36]. Pelvic lymph node metastases were confirmed in all of these patients. 4 patients (FIGO stage: IB1 in 3, and IIB in 1 patient) received an adjuvant chemoradiation. All patients had an abdominal radical hysterectomy and a pelvic lymphadenectomy. Prior to therapy, a chest X-ray and abdominal ultrasound was performed to exclude distant metastases.

### Imaging

For each patient, two consecutive treatment planning CT scans (CT scanner LightSpeed^® ^from GE Healthcare, General Electric Company, NYSE; GE), from the diaphragm to the trochanter minor with a slice thickness of 3.75 mm. were performed. The CT was performed with a belly board in prone position (PP), and with a head rest, a knee and ankle fixation in the supine position (SP). No instructions for bladder or rectum filling were given. The CT scans were acquired during a period when both scans were routinely carried out for patients with a high risk for paraaortic metastases, in order to switch the treatment technique easily if paraaortic metastases were histologically confirmed. Intravenous contrast mediaum (Xenetix 350^®^) and a vaginal tampon soaked in contrast medium were used to aid the delineation; oral contrast media was not used obligatorally.

### Target volumes and organs at risk

According to the recommendations of the International Commission on Radiation Units and Measurements Reports (ICRU) 50 and 62, target volumes and organs at risk were delineated in all axial CT slices [37,38]. For definitive treatment, the gross tumour volume (GTV) was defined as the macroscopic tumour, including the cervix with visible tumour extension and the corpus uteri. The clinical target volume (CTV) was defined by adding 5 mm to the GTV. Additionally, the external, internal and common iliacs and the presacral lymph nodes were included according to the RTOG recommendations [39]. For postoperative treatment, the CTV included all regions of potential microscopic disease: the surgical bed, regional lymph node areas (common, external and internal iliacs and the presacral region), and the vaginal cuff. The planning target volume (PTV) was outlined as the CTV plus 1 cm in all directions. The caudal field border was at the obturator foramen, the upper field border was individualized on the basis of the patient's anatomy to include the common iliac lymph nodes [40]. The bladder, the rectum (sigmoid to anus) and the small bowel (whole peritoneal cavity without lymph nodes, muscles and organs other than small bowel) were outlined as organs at risk. The delineation of the small bowel exceeded the upper and lower border of the PTV by 2 slices to generate comparable volumes. All contours were done by one investigator (C.S.) and review by the senior radiation oncologist (S. M).

### Dose Prescription and planning parameters

The Eclipse Planning Software (Version 7.3.10, Varian, Palo Alto, CA) was used to generate IMRT plans for SP and PP. The prescribed target dose was 5 × 1.8 Gy per week, to a total mean dose of 50.4 Gy (D_mean_). Treatment was performed on a linear accelerator (Clinac 2300CD, Varian, Palo Alto, CA) with 20 MV photon beams. Planning parameters for the PTV were set to minimize the amount receiving < 95% of the prescribed dose and the amount receiving > 105% (52.9 Gy) of the prescribed dose. The second highest priority was given to the sparing of small bowel. Inverse planning input parameters are shown in table 1. Constraints were applied as starting parameters and changed individually for each patient during optimisation. A help structure was generated by applying a 2 cm ring around the PTV and was used for normal tissue sparing adjacent to the PTV to achieve higher dose conformity.

**Table 1:** Planning parameters

Structure	Volume (%)	Gy	Priority
**Body**	0	54	500-800

**PTV**	0	52.9	400
	
	20	50.4	200
	
	80	50.4	200
	
	100	47.9	400

**Help**	0	49	100

**Small Bowel**	0	50.4	100
	
	10	43	150
	
	20	33	150

**Bladder**	30	50	100

**Rectum**	25	50	100

Inverse planning starting parameters for PTV and for organs of risk.

### Radiation Technique

Treatment planning and the DVH analysis was done with the Eclipse Planning Software (Varian Medical Systems, Palo Alto, CA). IMRT plans were generated based on a seven beam arrangement with beams at 45/90/115/180/245/280/320 degrees for SP and 0/40/80/115/235/270/320 degrees for PP, as is routinely applied at our clinic. The sliding window technique was used. Although patients also received brachytherapy, for this analysis, only external beam irradiation has been taken into account.

### Dose Volume Histogram Analysis

DVH parameters for the target volume and critical normal tissues were analysed, and the PTV95% (volume of PTV receiving 95% of the prescribed dose) and the D1% (highest dose delivered to 1% of the PTV) was calculated. The target conformity was calculated according to ICRU reports 50 and 62 (Conformity Index, CI) [37,38] and according to van't Riet and colleagues (Conformity number, CN) [41]. For all IMRT plans and patients set-up positions, the relative volumes (%) of small bowel, rectum and bladder were evaluated at 10 Gy (V10), 20 Gy (V20), 30 Gy (V30), 40 Gy (V40), 45 Gy (V45), and 50.4 Gy (V50.4). The average volumes (cc) and the mean dose (D_mean_) for the PTV and the organs at risk were measured. Dosimetric parameters were compared by the non-parametric Wilcoxon exact signed rank test (SPSS 15.0, Inc., Chicago, IL). Statistical significance was assumed for p ≤ 0.05.

## Results

### Target Volume

The mean volume of the PTV was 1227.0 cc (1110.8 - 1368.7 cc, standard deviation (STD) ± 66.6) for SP and 1369.4 cc (1085.4 - 1703.1 cc, STD ± 222.1) for PP (p = 0.6). D_mean _was 50.4 Gy in PP and SP, respectively. The mean volume of PTV95% was 97.0% (STD ± 1.2) for SP and 97.6% (STD ± 0.8) for PP. The mean D1% was 52.9 Gy (STD ± 0.2) and 52.8 Gy (STD ± 0.2) for SP and PP, respectively. The PTV that received 110% of the prescribed dose was < 0.01% in both groups. Conformity of IMRT plans for PP and SP IMRT gave similar results (CI: 1.13, STD ± 0.08 vs. 1.11, STD ± 0.06; CN: 0.85, STD ± 0.05 vs. 0.86, STD ± 0.05). All parameters did not reach statistical significance.

### Rectum

**Figure 1:**
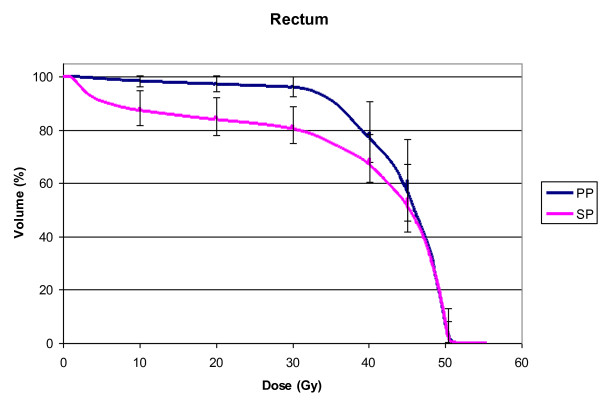
**DVH for Rectum**. Mean DVH of the rectum in SP (pink) compared to PP (blue). Error bars indicate the standard deviation (STD).

For the SP group, the delineated rectal volume ranged from 48.3 to 94.2 cc (mean volume 71.2 cc, STD ± 18.2) and for the PP group from 52.8 to 174.5 cc (mean volume 96.8 cc, STD ± 34.9; p = 0.08). In PP, a larger rectal volume was covered by the V10 to V40 (p ≤ 0.01, Figure 1). Neither the V45 nor the V50.4 or D_mean _(SP: 39.4 Gy, STD ± 3.5; PP: 40.3 Gy, STD ± 12.5; p = 0.3) showed a statistically significant difference (Table 2).

**Table 2:** DVH statistics

	Small bowel		Rectum		Bladder	
	PP	SP	PP	SP	PP	SP
**Volume (cc)**	1686.1 ± 368.7*	1250.6 ± 283.0	96.8 ± 34.9	71.2 ± 18.2	137.0 ± 93.4	143.9 ± 98.3
**D mean (Gy)**	25.9 ± 3*	30.5 ± 4.5	40.3 ± 12.5	39.2 ± 3.5	43.1 ± 2.8	44.2 ± 2.7
**V10 (%)**	91.5 ± 4.7	92.0 ± 10.5	98.6 ± 2.1	88.5 ± 6.6 *	100.0 ± 0	99.9 ± 0.4
**V20 (%)**	60.4 ± 11.0 *	76.4 ± 15.1	97.6 ± 2.9	85.2 ± 7.2 *	99.1 ± 1.9	98.8 ± 3.7
**V30 (%)**	32.7 ± 9.5 *	50.4 ± 13.9	96.5 ± 3.7	81.9 ± 7 *	92.1 ± 7.9	95.5 ± 7.4
**V40 (%)**	18.2 ± 5.8 *	28.4 ± 7.8	79.4 ± 11.2	69.5 ± 8.9 *	72.3 ± 15.0	77.4 ± 14.6
**V45 (%)**	13.7 ± 4.5 *	20.3 7 ± 5.5	61.3 ± 15.3	54.6 ± 12.7	53.3 ± 12.8	56.7 ± 15.0
**V50.4 (%)**	2.5 ± 1.2 *	4.0 ± 1.1	4.2 ± 4	4.6 ± 8.3	4.0 ± 4.6*	12.9 ± 16.1

Summary of DVH statistics for SB, rectum, bladder in PP and SP for both IMRT plans, mean values ± STD. * p ≤ 0.05 Wilcoxon signed rank test (exact) SPSS V15.

### Bladder

The bladder volume displayed a highly individual range for both positions. In SP, the bladder volume ranged from 70.7 to 417.7 ml, with a mean value of 143.9 ml ± 98.3 (STD), and from 70.2 to 395.2 ml, with a mean of 137.0 ml ± 93.4 (STD) (p = 0.6) in PP. The dose-volume histogram for the bladder was significant better in PP at V50.4 (p = 0.03). At V10, V20, V30, V40 and V45, no significant differences were detected (Table 2). D_mean _for SP was 44.2 Gy ± 2.7 (STD) and 43.1 Gy ± 2.8 (STD) (p = 0.7) for PP.

### Small Bowel

**Figure 2:**
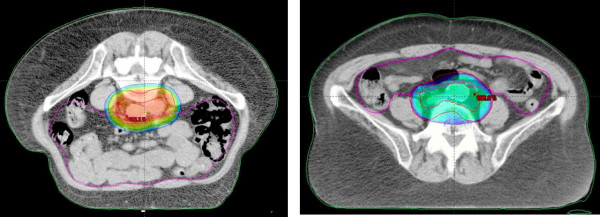
**a and b - Expansion of peritioneal cavity**. Small bowel movement in PP (left) and SP (right) for the same patient.

**Figure 3:**
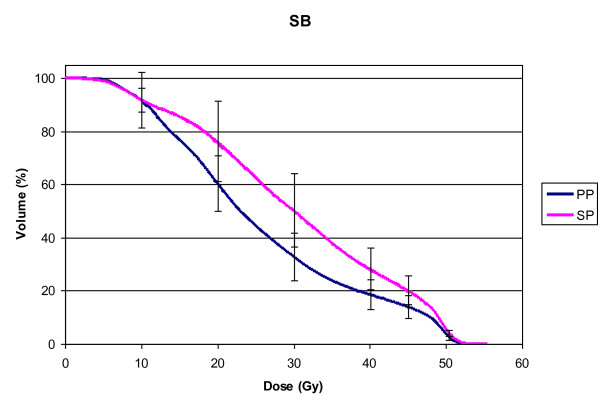
**DVH for small bowel**. Mean DVH of the small bowel: Supine position (SP, pink) compared to prone position (PP, blue). Error bars indicate the STD.

The small bowel volume varied from 683.8 to 1825.9 cc (mean 1250.6 cc, STD ± 283.0) for SP and from 1193.4 to 2443.9 cc (mean 1686.1 cc, STD ± 368.7) for PP. Statistically significant larger volumes of small bowel were found in PP (p = 0.01). Figure 2a. illustrates the expansion of the peritoneal cavity through the belly board in PP resulting in an anterior movement of the small bowel for these patients. The analysis of the pooled dose-volume histograms showed a significant decrease of the small bowel volume at V20, V30, V40, V45 and V50.4 in favour of the PP (p < 0.05, Table 2, Figure 3). D_mean _was 25.9 Gy vs. 30.2 Gy for PP and SP (p = 0.049), respectively.

## Discussion

Due to an overlap of target structures (lymph nodes) and organs at risk, there is a considerable rate of gastrointestinal and genitourinary morbidity in patients with cervical cancer undergoing pelvic irradiation [10]. With a platinum based chemoradiation, even a radiogenic total necrosis of the uterus is possible [42]. The risk of developing treatment related side effects depends strongly on the delivered dose, the irradiated volume, and any previous pelvic or abdominal surgery [11-17,43]. In the prone position, decreased dose to the small bowel was achieved by using bowel displacement devices [18-23]. The use of IMRT in clinical routine might decrease the risk for acute and late toxicity in patients after pelvic or paraaortic irradiation with comparable outcome [28,29,33,34,44-47]. Although IMRT and treatment in prone position on a belly board holds potential for decreased therapy related gastrointestinal toxicity, the implication of the patient's position when using IMRT has not been systematically investigated. In the 2-D era, Letschert and colleagues [16] found a correspondence between the risk for chronic diarrhoea and malabsorption and the amount of small bowel volume irradiated, but there was no correlation with bowel obstruction. In rectal cancer patients with postoperative pelvic radiotherapy (50 Gy), the risk for chronic diarrhoea and malabsorption after 5 years was 42% if the small bowel volume was above 328 cc vs. a risk of 31% for volumes < 77 cc [17]. We could reduce the mean small bowel volume receiving 50.4 Gy to 42 cc (2.5%) and 50 cc (4%) and the V45 to 231 cc (14%) and 254 cc (20%) for PP and SP, respectively. Roeske and colleagues associated acute bowel morbidity with small bowel volumes receiving ≥ 45 Gy [29]. Portelance and colleagues [28] showed a significant reduction of the small bowel volume receiving 45 Gy or more with IMRT compared to a 2- and 4-field-technique. Heron and colleagues [26] found a 52% decrease of the small bowel volume by IMRT.

Few publications addressed the issue of IMRT and the patients' position [18,48]. In a planning study performed by Adli and co-workers [48], DVH parameters for two different IMRT techniques (limited arc vs. extended arc) in prone versus supine treatment positions of 16 gynaecologic cancer patients (7 postoperative, 9 definitive) were compared. In the present study, we mixed patients with definitive and adjuvant irradiation. George et al. did not see a statistical difference for small bowel sparing in gynaecologic patients with either definitive or adjuvant IMRT treatment in supine position [25]. We therefore pooled patients with primary or postoperative treatments in this planning study, as has been done by others [48].

The prescribed total dose in the study by Adli was 45 Gy, where the small bowel was defined as all individual loops. They observed an anterior movement of small bowel for patients in PP, as we did. A dosimetric benefit was found for PP irrespective of the IMRT techniques. They concluded that the magnitude of small bowel sparing did not merely depend on the prone treatment position, but on the specific IMRT technique used. In our study, we did observe a larger "small bowel" volume (mean 436 cc; +35%) in PP, evolving from an expansion of the peritoneal cavity not basically due to more small bowel loops but a widening of the space between the loops though the opening in the belly board and an cranial movement of small bowel in supine position. Our small bowel volume for the PP group at 45 Gy and 50.4 Gy was 13.7% and 2.5%, as compared to the data from Adli and colleagues who saw 12.5% and 10% (45 Gy) and 5% and 6.6% (50 Gy) for limited arc or extended arc, respectively [48]. Our prescribed dose was slightly higher (D_mean _50.4 Gy), but the sparing of small bowel at V50 was considerable better. The sliding window technique, the 20 MV photon beam and the different contouring of the small bowel might contribute to this volume reduction. Interestingly, Adli and colleagues reported on dose inhomogeneities of up to and over 130% of the prescribed dose for both IMRT techniques.

No significant dosimetric benefit was seen between PP and SP in a more recently published study by Beriwal and colleagues [18]. They analysed 47 patients with endometrial cancer treated with IMRT. 21 patients were treated in prone and 26 patients in supine position. An inter-individual dosimetric and toxicity comparison was performed. The patient cohort was inhomogeneous, 8 patients had pelvic and paraaortic radiotherapy (4 in PP, 4 in SP), 7 patient had chemoradiation (4 in PP, 3 in SP) and the prescribed D_mean _ranged form 45-50.4 Gy. All patients received 10 Gy HDR brachytherapy to the vaginal-cuff. Small bowel volumes (defined as the peritoneal surface) at 45 and 50 Gy were remarkably low in PP and SP (V45: 5.8% and 6%; V50: 1.4% and 1.2%). The IMRT treatment was well tolerated and only one single Grade 3 chronic gastrointestinal toxicity was reported. The authors found no correlation between gastrointestinal morbidity and dosimetric parameters among the opposed set-up positions after a median follow up of 19-20 months. They therefore concluded that a longer follow up is needed to detect any existing differences between the two approaches.

Our aim was to compare the best IMRT 7-field standard technique at our department for the prone position and the supine position. Many factors influence the magnitude of dose reduction to the organs at risk. One key issue is the target volume definition. Large amounts of small bowel are located in the upper pelvic region, around the upper iliac external and common iliac lymph nodes. Our target definition was comparable to those of other authors [25-29]. Furthermore, the contouring of the small bowel is not standardized. Some author's delineated single loops [25,27,48], others preferred to delineate the whole peritoneal cavity [18,26,28,46] as we did. In our study, we did observe a larger "small bowel" volume (mean 436 cc; +35%) in the prone position, evolving from an expansion of the peritoneal cavity, due not basically to more small bowel loops in the peritoneal cavity but to a widening of the space between the loops through the opening of the belly board. A similar cranial displacement of the small bowel had been observed by Das and colleagues for the prone position [19]. Additionally, the amount of bladder filling might have an impact on uterus motion and consecutively on small bowel motion. Georg and colleagues [25] showed that bladder size correlated with small bowel sparing for definitive pelvic radiotherapy with IMRT in the supine position, but not in patients who had a hysterectomy. In the present study, no emphasis was placed on bladder or rectum filling, and in addition, low planning priority was given for sparing of these organs at risk. Due to these issues of study design, no significant differences in the sparing of the rectum and bladder (exception: V50.4 Gy in PP) could be found. One issue raised against the prone position is the possibility that it is a less reliable and less stable treatment position than the supine one. New data support that the patients' set-up in prone position harbours larger systematic errors, but the set-up in supine position harbours a larger random error [24]. We recommend performing a frequent or even daily on-board imaging with a KV (kilovolt) or MV (megavolt) CT to provide the best possible reduction of set-up errors and treatment accuracy when using either position.

## Conclusion

In this dosimetric study, we demonstrated that pelvic IMRT in prone position on a belly board seems to be a useful tool to reduce small bowel volume at a dose ≥20 Gy whilst providing similar target coverage and target conformity for patients with cervical cancer. Despite this, new evidence supports a comparable set-up error for the prone and supine treatment positions. We recommend frequent onboard imaging with KV or MV CTs to assure optimal set-up accuracy. Nevertheless, only outcome studies will show if the dosimetric differences in small bowel sparing will lead to decreased acute and late gastrointestinal toxicity.

## Competing interests

The authors declare that they have no competing interests.

## Authors' contributions

CS did the collection and assembly of data, data analysis and interpretation, and the manuscript writing. SM carried out conception and design, and manuscript writing. YK and TM optimised the IMRT plans. MK and UJ carried out data interpretation. CK and AS helped with the collection of data and to draft the manuscript. VB helped with the interpretation of the data and to draft the manuscript. All authors read and approved the final manuscript.
